# Working without a blindfold: the critical role of diagnostics in malaria control

**DOI:** 10.1186/1475-2875-7-S1-S5

**Published:** 2008-12-11

**Authors:** Mark D Perkins, David R Bell

**Affiliations:** 1 Foundation for Innovative New Diagnostics (FIND), 1 Ave Louis Casai, CH-1216, Geneva, Switzerland; 2 World Health Organization – Regional Office for the Western Pacific, P.O. Box 2932, Ermita 1000, Manila, Philippines

## Abstract

Diagnostic testing for malaria has for many years been eschewed, lest it be an obstacle to the delivery of rapid, life-saving treatment. The approach of treating malaria without confirmatory testing has been reinforced by the availability of inexpensive treatment with few side effects, by the great difficulty of establishing quality-assured microscopy in rural and resource-poor settings, and by the preeminence of malaria as a cause of important fever in endemic regions. Within the last decade, all three of these factors have changed. More expensive artemisinin combination therapy (ACT) has been widely introduced, simple immunochromatographic tests for malaria have been developed that can be used as an alternative to microscopy by village health workers, and recognition of the health cost of mismanaging non-malarial fever is growing. In most of the world a small fraction of fever is due to malaria, and reflex treatment with ACT does not make medical or economic sense. Global malaria control efforts have been energized by the availability of new sources of funding, and by the rapid reduction in malaria prevalence in a number of settings where bed nets, indoor residual spraying with insecticides, and ACT have been systematically deployed. This momentum has been captured by a new call for malaria elimination. Without wide implementation of accurate and discriminating diagnostic testing, and reporting of results, most fever will be inappropriately managed, millions of doses of ACT will be wasted, and malaria control programmes will be blindfolded to the impact of their efforts.

## Introduction

Malaria control has recently seen a long-overdue resurgence in interest and funding, a belated recognition that malaria is still dominating the lives and health of millions, and holding whole populations in poverty. In places where these renewed efforts have been systematically applied, insecticide-treated bed nets, indoor residual spraying, and artemisinin-based combination therapy (ACT) have demonstrated their capacity to reduce both incidence and mortality from malaria. For the first time in decades, the idea of elimination of malaria as a public health problem, and perhaps complete interruption of transmission across whole regions, is being considered. However, although modern prevention and treatment tools for malaria are shown to be effective, long-term success in controlling malaria, and in reducing the morbidity and mortality of fever in the tropics, will require a fundamental change in the way fever is managed, and in the specificity with which malaria care is allocated. This article argues that the role of diagnosis must be prioritized, both for case management and for surveillance, and that progress toward malaria elimination will only increase the need for good diagnostic information.

## Background

Most causes of fever in the tropics are transient, non-fatal illnesses. In the latter half of the 19^th ^century, it was discovered that many of the infectious causes of fever that did have the potential to be fatal, including malaria, leishmaniasis, tuberculosis, sleeping sickness and others, were detectable by microscopy. For one of these, malaria, there was a relatively early pharmacological intervention thanks to the long history of quinine (cinchona) use in the Americas. The utility of microscopy in tropical fevers, and the availability of life-saving treatment for malaria, led to the wide advocacy of microscopy-based case management as the standard of care. When microscopy was found to be untenable in many parts of the world, due to the massive effort and resources required to maintain such a service in close proximity to the rural and poor populations widely at risk, syndromic management, classifying all 'malaria-like' fevers as malaria, again became the de facto standard of practice.

For the last three quarters of a century then, since the discovery and development of chloroquine in the 1930s and 40s, the medical community has treated most fevers in malaria-endemic countries as malaria, forgoing the diagnostic process. This practice has been codified into national and international recommendations and training manuals for health workers, especially for fever in children. The common teaching has been 'fever equals malaria unless proven otherwise'.

Clearly many lives have been saved by pushing for rapid, even community or home-based access to antimalarial therapy, regardless of diagnostic testing. In the many communities in which malaria has accounted for the majority of potentially fatal causes of fever, it has been hard to imagine any other approach, given the poor performance and relative unavailability of microscopy [1-6].

Over the past decade, though, a number of important changes have taken place in the epidemiology and control of malaria and in the diagnostic techniques available, that dramatically alter the balance of rational action in favour of parasite-based diagnosis over blind therapy of fever with anti-malarial drugs. Specific diagnosis of malaria is now not only possible, but necessary, and scaled up malaria control efforts, including elimination plans, must include expanded and quality assured use of parasite-based diagnostic testing and reporting of results.

### Overdiagnosis of malaria and fever mismanagement

Unfortunately, the clinical presentation of malaria is highly variable and overlaps with that of a number of other common illnesses, including pneumonia, which are associated with significant morbidity and mortality [7,8]. Attempts to develop clinical scoring systems with high predictive values have largely been unsuccessful [9-12], and health workers without access to tools for parasite-based diagnosis often manage most or all fevers as malaria. This practice continues to be included in medical training and in national treatment guidelines [13]. As summarized by Amexo and colleagues [14], however, presumptive management of fever as malaria results in significant overdiagnosis, even in high-risk areas. In many settings, especially where malaria is seasonal or where intensive disease control efforts are implemented, a small minority of febrile patients may be parasitaemic [15-18]. The 2008 World Malaria Report estimates well below a third of fevers in endemic areas of Africa are due to malaria, and much less again in other regions. In India, for instance, slide positivity rates, based on approximately 100 million slides examined, are about 2% [18]. Even when WHO criteria for severe malaria are met, an important fraction of patients may be found to have an alternative cause of fever on post-mortem or other careful investigation [19,20]. Unfortunately, not enough is known about the causes of non-malarial fevers in the tropics.

During a period when inexpensive drugs such as chloroquine were available, widespread overtreatment for malaria was accepted as a means to improve treatment coverage and decrease mortality. With rising drug resistance, national malaria control programmes are moving to more expensive and more effective artemisinin combination therapy (ACT), and the cost of drugs wasted on treatment of non-malarial fever become substantial.

**Figure 1:**
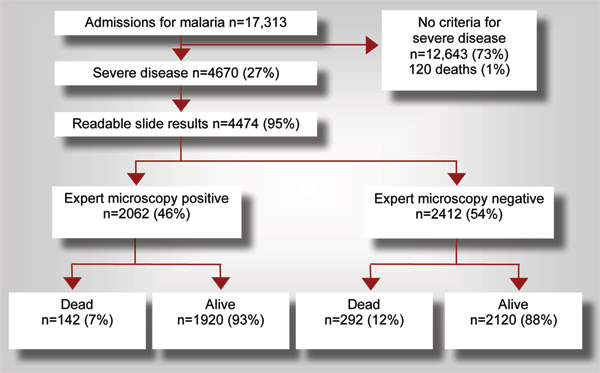
High mortality at 10 Tanzanian hospitals among patients admitted with severe disease and treated for malaria. (Adapted from [22]).

Malaria misdiagnosis and subsequent mis-management has individual as well as societal repercussions. At the individual level, misdiagnosis results in wasted resources on drug purchase, especially in the private sector, exposure to drug side effects, and most importantly morbidity due to improper management of the true cause of fever. Early treatment with effective drugs is vital in acute respiratory tract infections, meningitis, and other differential diagnoses of malaria, in the same way that early, appropriate and effective treatment is fundamental to reducing malaria case fatality rates (Figure 1) [21]. Due to the cost of drugs and of complications from inappropriately managed illness, and to the limitations in access to good microscopy services in remote and resource-poor locations, impoverished individuals are disproportionately affected by malaria misdiagnosis [14]. At a societal level, mismanagement of fever erodes patient's faith in the health system, wastes drug resources, augments drug pressure toward resistance [22], and contributes to ignorance of the true causes of illness in these populations. In the face of this, it should be disturbing that less than 20% of suspected malaria cases receive a confirmatory diagnosis in 75% of African countries [18].

One of the drivers of presumptive treatment is fear of rapid mortality of untreated malaria, especially in young children. Increasing evidence suggests that where accurate parasite-based diagnosis is present, febrile children with negative malaria examinations may safely be cared for without antimalarial treatment [23,24]. A carefully performed study in Uganda examined whether, in a setting of low to moderate transmission, microscopy-directed therapy would result in excess mortality from unrecognized and untreated malaria. Of 2,359 medical visits by children <10 years of age presenting with fever, 1,608 (68%) were microscopy-negative and were not given anti-malarial drugs. During seven days of follow-up, only 13 (0.8%) were subsequently diagnosed with malaria. There were no deaths, and no episodes of severe malaria [15]. Similarly, Ngasala and colleagues found that microscopy-based treatment was safe and effective in children under five years old, even where less expert microscopy was used for screening [25]. Modeling studies suggest that even at high parasite prevalence the benefits of improved management of non-malarial illness make parasite-based diagnostic methods cost-effective [26]. WHO is currently developing evidence-based recommendations in this area.

### Development of rapid diagnostic tests (RDTs)

Microscopy services for the diagnosis of malaria are not widely available, especially at the community level where most urgent care takes place. Microscopy infrastructure is cumbersome to develop, requires trained and motivated staff, well-maintained equipment, and a well-executed quality assurance system. In the hands of many microscopists in low-income settings where training, equipment and reagents may be substandard, the accuracy of microscopy is poor [1-6]. A 2006 study in Kenya found routine microscopy to be only 69% sensitive and 62% specific when compared with expert microscopy [3]. While standards vary and microscopy remains invaluable for diagnosis, parasite quantitation and treatment monitoring where good standards can be maintained, ensuring the quality of microscopy as a system-wide approach has proved beyond the capability of most malaria control programmes.

For these reasons the development, in the early 1990s, of lateral flow immunochromatographic tests that could detect malaria parasite antigens in a fingerprick blood sample was a major advance [27,28]. These rapid diagnostic tests (RDT) are based on the ability of monoclonal antibodies to bind to parasite antigens in lysed blood and immobilize them along a defined line on nitrocellulose for detection with a colored label (most commonly colloidal gold). All existing commercial RDTs target one or more of well characterized Plasmodium protein targets: histidine rich protein 2 (HRP2), parasite lactate dehydrogenase (pLDH), or aldolase (see Table 1). HRP2 is present in *Plasmodium falciparum *only, whereas pLDH and aldolase, with some conserved and some variable epitopes, are present in all four major species causing human malaria. RDTs have the obvious advantage of requiring less training, being easily performed in remote or village settings, and putting most of the quality control responsibility in the hands of the manufacturer instead of the user.

**Table 1:** Target antigen combinations in commercially available RDTs. Adapted from [34].

**RDT type**	**Target antigens**	**Possible results**
I	HRP2	No Pf
		Pf
		Invalid

II	HRP2	No malaria
	Aldolase (all species)	Pf or mixed
		Pv, Po, and/or Pm
		Invalid

III	HRP2	No malaria
	pLDH (all species)	Pf or mixed
		Pv, Po, and/or Pm
		Invalid

IV	pLDH (falciparum-specific)	No malaria
	pLDH (all species)	Pf or mixed
		Invalid

V	pLDH (falciparum-specific)	No malaria
	pLDH (vivax-specific)	Pf
		Pv
		Pf + Pv
		Invalid

VI	HRP2	No malaria
	pLDH (falciparum-specific)	Pf or mixed
	pLDH (vivax-specific)	Pf + Pv, +/- Po and/or Pm
		Pf +/- Po and/or Pm
		Pv +/- Po and/or Pm
		Po and/or Pm
		Invalid

VII	Aldolase (all species)	No malaria
		Pf, Pv, Po and/or Pm
		Invalid

**Figure 2:**
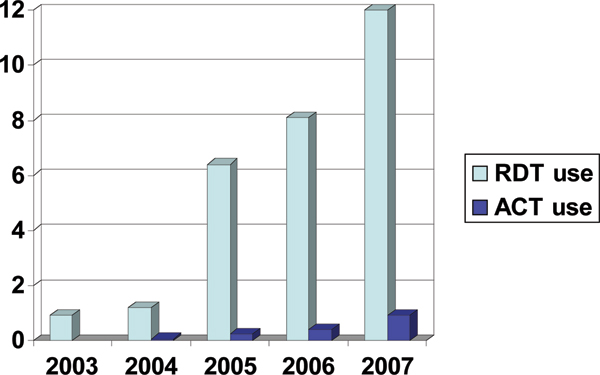
Scaled-up use of ACT and RDTs in India, in millions.

These tests are in relatively wide use in many South American and Asian countries, where use has been scaled up, as seen in Figure 2 below from India, to accompany ACT [29]. Use is now increasing in a number of African countries, such as Ethiopia, Zambia, Uganda and Nigeria, after specific allocation of funds for RDT purchase in proposals to the Global Fund Against AIDS, TB, and Malaria (GFATM) and other funding sources. Global sales of malaria RDTs are thought to surpass 70 million tests a year. From a single manufacturer in 1993, there are now dozens of diagnostics companies manufacturing or re-branding malaria RDTs. There are several sources of monoclonal antibodies against the common antigen targets, and companies making lateral flow tests for other indications such as pregnancy testing, screening for illicit drugs or diagnosis of infectious diseases can, without much difficulty or expense, add malaria to their test portfolio.

The profusion of malaria test manufacturers, many of them small, combined with their difficulty obtaining reference clinical materials from well-characterized malaria patients and the lack of biologic reference standards, has resulted in significant variability in the quality of tests being manufactured. Dozens of diagnostic trials of RDT performance have been carried out, and several reviews published [30-33]. Variability in trial design, in population selection, in reference methods, and in handling and storage of the RDTs make it difficult to draw robust conclusions about the performance and accuracy of specific RDTs tested in these studies, and impossible to distinguish between potential problems with the quality of the tests themselves and factors specific to the trial.

Many RDTs *have *been shown to perform well, detecting over 90% of malaria cases, including those with relatively low parasite density (200 parasites/μl). Several RDTs distinguish between falciparum and non-falciparum malaria, and in ideal conditions bring the power of reliable expert microscopy (detection, species identification, and to a much lesser extent, quantitation) and put it in the hands of village health workers. Unfortunately, though these tests have been shown to be useful, cost-effective [26], and safe in the direction of antimalarial therapy [23,24], health care workers frequently ignore results, either because of ingrained treatment habits, pressure to treat from patients and family members, or doubt about the accuracy of the RDT results [16]. Clearly, if RDTs are going to shift the global paradigm of fever management from reflex antimalarial treatment to diagnostic-directed treatment, systems will need to be in place to: 1) ensure the confidence of the health worker in the performance of the diagnostic test [34], and 2) provide capacity to appropriately and effectively manage the illnesses of RDT-negative patients [14,35].

### Assuring the quality of malaria RDTs

Over the past several years, a three-pronged programme of quality assurance for malaria RDTs being used in the public sector has been put in place. Led by WHO, FIND (Foundation for Innovative New Diagnostics) and multiple partners, these activities are intended to address uncertainties both about the quality of manufacture as well as the stability and performance of RDTs post-purchase.

One part of this programme involves assembling a restricted list of high-quality rapid tests for purchase through the public sector. To be on this list, tests must be manufactured under ISO-13485:2003 certification and must show good performance when tested against a panel of reference materials. To evaluate performance, a bank of highly characterized reference materials has been established at the US CDC that contains cultured parasites, recombinant proteins, and large numbers of aliquots of blood from malaria-infected patients whose blood is collected under protocol and then diluted to fixed and clinically-relevant concentrations of parasites. Results from the first round of this testing should be available in January 2009, and are expected to direct public sector purchasing toward only those tests showing good performance and temperature stability.

A second part of the QA programme allows countries or agencies purchasing RDTs for public sector use to submit samples of purchased lots for testing after arrival of the products. This ensures both that the lot purchased meets performance and quality standards, and that the tests maintained accuracy despite temperature and humidity stresses encountered during shipment [36]. With this capacity now in place, there is no reason that sub-standard batches of RDTs should now be delivered to health workers in the field.

The third arm of the WHO/FIND QA process for RDTs is to create positive control wells (PCW), composed of recombinant antigens at concentrations intended to give a weak-positive result, that should allow the village health worker or other care provider to test an RDT from any box before use, ensuring that the kit has not degraded during storage and transport, and bolstering confidence that the results of testing may be used to direct therapy. This development promises to greatly improve on the current necessity to perform comparative microscopy at sentinel sites to monitor RDT field performance. Prototype PCWs, designed for long-term stability, will be evaluated in field studies in the first quarter of 2009.

### Diagnostics in the malaria elimination campaign

Now 40 years after the failure of the first Global Malaria Eradication Campaign [37], there are new calls for malaria elimination, and in a few settings advanced degrees of control have already been accomplished. At the recent 2008 Millennium Development Goals Malaria Summit, an ambitious new Global Malaria Action Plan was endorsed and nearly $3 billion committed towards reducing the number of malaria deaths to near zero by 2015.

A drive towards malaria elimination will increase the need for broad use of quality assured diagnostics for malaria, and possibly the development of novel assays to address specific needs such as the sensitivity at low parasite densities and minimal invasiveness required for population-based surveys for parasite reservoirs. The imperative for parasitologic diagnosis will be dramatically increased where malaria incidence falls, and where reflex treatment of fever with antimalarial drugs would be ineffective and even harmful in the vast majority of cases.

**Figure 3:**
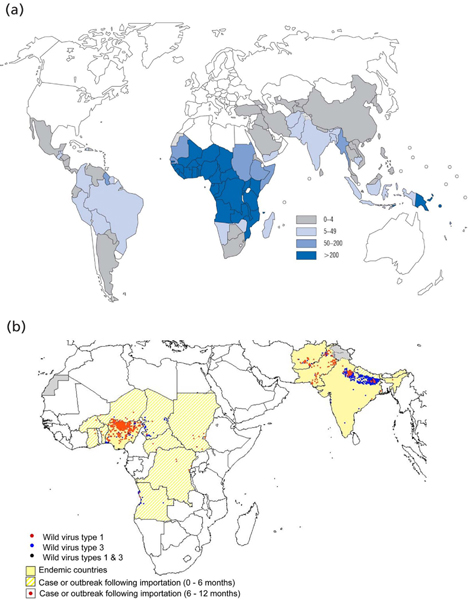
As malaria moves towards elimination and funding agencies require clearer feedback on progress, malaria reporting will need to move towards more real-time and geographically-refined incidence reporting. This will require the vast majority of malaria-like fevers to be diagnosed using parasite-based diagnosis to confirm presence or absence of malaria parasites. At present, malaria reporting is at a relatively gross level. **(a) **Map of estimated malaria incidence per 1000 population, 2006 (Source: World Malaria Report, 2008), compared with some other diseases with well-developed case finding and reporting systems. **(b) **Map of wild poliovirus infections detected 29 Oct 2007 to 28 Oct 2008.

More important perhaps, is the role of routine malaria testing as a surveillance method. Mapping malarial cases will be critical to understanding the effectiveness and impact of different control policies that are being implemented. Though this is currently done through testing at sentinel sites or in large population-based surveys, much more accurate and real-time information can be gathered through the development of reporting systems that capture the results of RDT testing at village level. Currently, very few RDT results are recorded and transmitted back to centralized levels of the health system in a way that could serve surveillance needs. For both HIV and tuberculosis, relatively robust systems are in place that capture the results of testing in peripheral settings and establish the basis for national reporting. Though this will be a greater challenge for malaria, recent developments, including the existence of RDTs, the strengthening of malaria services with a rapid increase in funding, and the drive toward elimination, make such planning feasible. System-wide reporting would also highlight outbreaks, and could serve as early harbinger of emerging drug resistance or increased insecticide resistance in mosquitoes. Elimination planning and monitoring will require faster and more accurate information about the incidence and distribution of disease than is currently available (Figure 3).

Ultimately, the Global Malaria Eradication Campaign died in the 1960s from donor fatigue. Elimination campaigns are costly and difficult, even when highly effective tools are available. Without clear and detailed evidence of the impact of donor spending on malaria case rates and geographic distribution, donors cannot be expected to continue to fund control campaigns. The Global Polio Eradication Initiative, spearheaded by WHO, Rotary International, the US CDC and UNICEF, has spent more money since it missed its goal of eradicating polio by the year 2000 than in the years leading up to that milestone. Such support would not have been possible without the kind of incidence mapping, such as that shown in Figure 3b, that gives donors and public health planners the information they need to be assured that the strategy employed is making an impact, and has clearly defined goals.

### New diagnostics

It is likely that if success in malaria control is obtained, it will lead to the need for, and development of, new diagnostic tests. Much of the large public sector market for malaria RDTs is served by tests that detect only *P. falciparum*, especially in Africa. Despite limitations due to antigen persistence after parasite death [38-40], and target antigen variability [41], tests detecting only HRP2 from *P. falciparum *are most commonly procured, as they are generally less expensive, may be more stable across temperature extremes [42], and tend to have a lower threshold of detection [43]. Detection of *P. falciparum *will guide treatment for cases most likely to progress to severe disease, and mono-infections with non-*P. falciparum *species are uncommon in much of sub-Saharan Africa. As ACTs are increasingly used, however, it is likely that non-falciparum malaria will form an ever-greater fraction of cases. This is already true in parts of Ethiopia, where the conventional ratio of 70/30 falciparum/non-falciparum recently reversed in some areas of the country. Public sector deployment of RDTs in Africa may increasingly shift away from falciparum-only tests. Ongoing research aims to develop more stable tests that allow species differentiation, against either the current targets or novel target antigens.

The sensitivity of tests for malaria may need to higher in areas where good malaria control is being effected than current testing allows. Microscopy and RDTs both become relatively insensitive at parasite densities below 100 parasites/μl. PCR studies have shown that low-level parasitaemia missed by RDTs and conventional microscopy can be uncovered with sensitive molecular methods. As individuals with low-level parasitaemia may act as reservoirs for transmission, their detection may be necessary to accelerate elimination. Various groups are working on the development of potentially highly sensitive assays that may be used in peripheral laboratories for detection of falciparum and non-falciparum malaria [44-46]. To eliminate malaria in areas with significant transmission of *P. vivax *or *P. ovale*, tests may also be required that detect hypnozoites to guide the use of 8-aminoquinolones (primaquine, terbinafine) that clear these liver stages and prevent relapse.

## Conclusion

Sustained malaria control will depend on the global capacity to accurately detect malaria and map its distribution. The specific detection of malaria parasites is now possible even at the village level with high quality rapid diagnostic tests. Driven by the extent of over-diagnosis and misdiagnosis of malaria when syndromic approaches are used, global efforts are underway to increase the utilization of parasite-based diagnosis, and to ensure the quality of tests that are used. Elimination efforts will not only increase the need for widespread RDT use, but may drive the development of new tests with enhanced performance. Implementation of the Global Malaria Action Plan [47], proposed regional initiatives towards elimination of malaria, and the reductions in mortality from malarial and non-malarial illness necessary to achieve Millennium Development Goals will require an increased emphasis on building systems for parasite detection as an integral part of malaria case and programme management.

## Competing interests

The authors declare that they have no competing interests.

## Authors' contributions

MP and DB both conceived and wrote the manuscript.
